# Unified Phase Criterion for Band Crossings in One‐Dimensional Topological Photonics

**DOI:** 10.1002/nap2.70329

**Published:** 2026-11-06

**Authors:** Jingxuan Song, Haiyi Liu, Changlin Sun, Pengfei Qi, Weiwei Liu

**Affiliations:** ^1^ Institute of Modern Opics, College of Electronic Information and Optical Engineering National Key Laboratory of Semiconductor Laser Nankai University Tianjin China; ^2^ Tianjin Key Laboratory of Micro‐Scale Optical Information Science and Technology Tianjin China; ^3^ Academy for Advanced Interdisciplinary Studies Nankai University Tianjin China

**Keywords:** band‐crossing points, Brewster angle, one‐dimensional photonic crystals, topological photonics

## Abstract

One‐dimensional photonic crystals (1DPCs) provide a simple and versatile platform for exploring topological photonics. Topological effects in these layered systems have enabled a range of applications in robust light confinement, lasing, modulation, and disorder‐tolerant optical devices. Among their underlying band features, band crossings are of particular interest because they represent critical degeneracies associated with topological phase transitions in periodic media. However, although band crossings in one‐dimensional topological photonics have been extensively studied, a unified theoretical criterion for their occurrence remains unclear. In this work, a unified phase criterion for band crossings that applies to both TE and TM polarizations from normal to oblique incidence was derived based on transfer‐matrix method and Bloch theory. Notably, for TM polarization, besides the generic band crossings satisfying the integer‐phase criterion, the Brewster condition enables additional crossings without the phase constraint, forming a straight trajectory in momentum space. These results establish a direct connection between classical interface optics and band‐crossing formation in periodic media, and provide a unified framework for understanding and engineering band crossings in layered photonic structures. These findings provide design guidelines for multilayer photonic devices: Brewster‐angle transmission improves fabrication tolerance, whereas polarization‐independent band crossings enable robust polarization‐insensitive optical responses.

## Graphical abstract

A unified phase criterion reveals the origin of band crossings in one‐dimensional photonic crystals. The accumulated propagation phases in constituent layers determine the emergence of degeneracies for both TE and TM polarizations. The Brewster condition is further identified as a special trajectory of TM band crossings, enabling disorder‐robust high‐transmission channels in multilayer photonic structures.

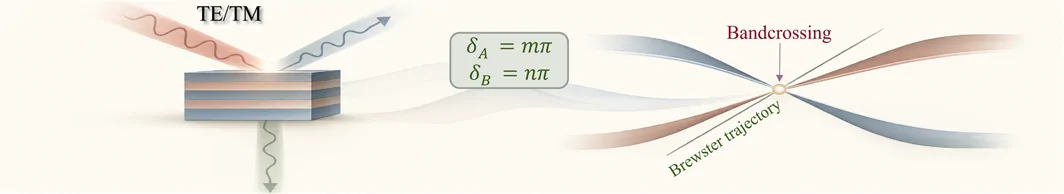

## 1 Introduction

Topology, originally introduced to characterize properties preserved under continuous deformations, has become a central concept in photonics [1, 2, 3, 4]. Compared with conventional photonic states, topological photonic states generally exhibit enhanced robustness against imperfections and disorder [4, 5, 6]. By combining band topology with the flexibility of optical platforms, topological photonics has provided a powerful framework for understanding and exploiting protected transport, interface‐state formation, and robust field localization [7, 8, 9, 10]. Among the various platforms of topological photonics, one‐dimensional photonic crystals are especially attractive because they are easy to fabricate and characterize experimentally [11, 12]. Despite their simple layered geometry, 1DPCs support rich topological physics [13] including Zak‐phase band characterization [14, 15], topological interface states [16, 17], and related topological phenomena [18, 19]. These features have enabled a variety of device applications, such as topological nanocavity lasers [20, 21], high‐speed electro‐optic modulation [22], and disorder‐tolerant filters [23, 24] and sensors [25, 26, 27, 28, 29, 30], based on topological interface states.

For one‐dimensional topological photonics states, band crossings are of particular importance because they serve as key signatures of topological phase transitions. In these systems, topological phase transitions are typically accompanied by band‐gap closing and reopening, which govern changes in band topology and the emergence of topological interface states [31, 32, 33]. From this perspective, band touching or band crossing at the critical point of the transition is not merely a spectral feature, but a direct indication of the underlying topological change [34]. Therefore, a systematic investigation of band crossings is essential for exploring a wider variety of topological states and for developing more topological photonic devices based on one‐dimensional photonic crystals [35, 36, 37, 38, 39].

Scientific community has experimentally investigated band crossings in one‐dimensional topological photonics under different polarizations and incidence conditions [8, 40]. Based on such band crossings, more complex topological states, including nodal‐line states and photonic Dirac nodal‐line states, have been realized on one‐dimensional photonic‐crystal platforms, highlighting the application potential of band crossings in topological photonics. Moreover, a prominent spectral signature of band crossings is the appearance of high‐transmission regions in the reflection spectra. Although the connection between band crossings and high‐transmission features in one‐dimensional topological photonics has only recently been highlighted, classical optics has long provided a well‐known high‐transmission condition for TM waves, namely the Brewster angle. This naturally raises several questions: What is the connection between TM‐polarized band crossings and the Brewster angle? For TE polarization, is there still a general mechanism for band crossing? Can a unified mechanism for band crossing for both TM and TE be established? Answering these questions is important for designing artificial metamaterials such as perfect angle‐selective transmission media [41]. Previous theoretical studies have also revealed rational optical‐path conditions associated with band degeneracies in one‐dimensional photonic crystals, particularly under normal‐incidence conditions [8]. Recently, Li et al. [16] investigated incident‐angle‐induced topological phase transitions in one‐dimensional photonic crystals and identified the Brewster angle as a TM‐specific critical transition angle, in addition to structure‐dependent critical angles shared by TE and TM modes. They further exploited these angle‐induced transitions to achieve TE–TM splitting of topological interface states. Nevertheless, a unified criterion for the formation of band crossings themselves, as well as a general interpretation of how the Brewster condition is embedded in the band‐degeneracy structure, remains to be established.

In this work, we investigate the formation mechanism of band crossings in one‐dimensional topological photonics using the transfer‐matrix method and Bloch theory. Starting from the band‐dispersion relation, we derive a general degeneracy condition for different incidence angles and analytically demonstrate that band crossings occur only when the propagation phases accumulated in both constituent layers are integer multiples of π.This unified phase criterion applies to both TE and TM polarizations from normal to oblique incidence, constrains the crossings to high‐symmetry points of the Brillouin zone, and naturally transit into linear Dirac‐cone dispersions near the degeneracy points. Additionally, the classical Brewster angle admits a new physical interpretation from this perspective: the Brewster angle corresponds to a set of isolated band‐crossing points that develops into an angle‐dependent crossing trajectory of band degeneracies in momentum space for TM polarization. These findings construct a direct connection between classical optical effects and band structures in periodic media, and deepens our understanding of degenerate‐state formation in layered photonic structures, which can provide practical strategies for engineering band crossings through polarization and incident‐angle control, offering a route to angle‐tunable Dirac dispersion and topological photonic devices. More broadly, the theoretical framework provides a platform for connecting conventional optical concepts with emerging topological functionalities in artificial electromagnetic metamaterials, exploring classical‐optics‐guided topological design in other layered and anisotropic photonic systems.

## 2 Results and Discussion

The one‐dimensional photonic crystal considered in this work is illustrated in Figure 1. The structure consists of two dielectric layers, denoted A and B, with refractive indices *n*_A_, *n*_B_, thicknesses *d*_A_, *d*_B_, and lattice period *a* = *d*_A_ + *d*_B_. These layered media are arranged periodically along the z direction and are regarded as infinite in the *x* and *y* directions, an assumption that is well justified when the transverse dimensions of the structure are much larger than the spatial extent of the incident optical field. Throughout this work, the constituent materials are assumed to be lossless and dispersionless. TE and TM‐polarized light are incident on the photonic crystal surface at a finite angle. The results presented in this work are calculated by the transfer‐matrix method and Bloch theory (see Supporting Information S1: Section S1, S2).

**FIGURE 1:**
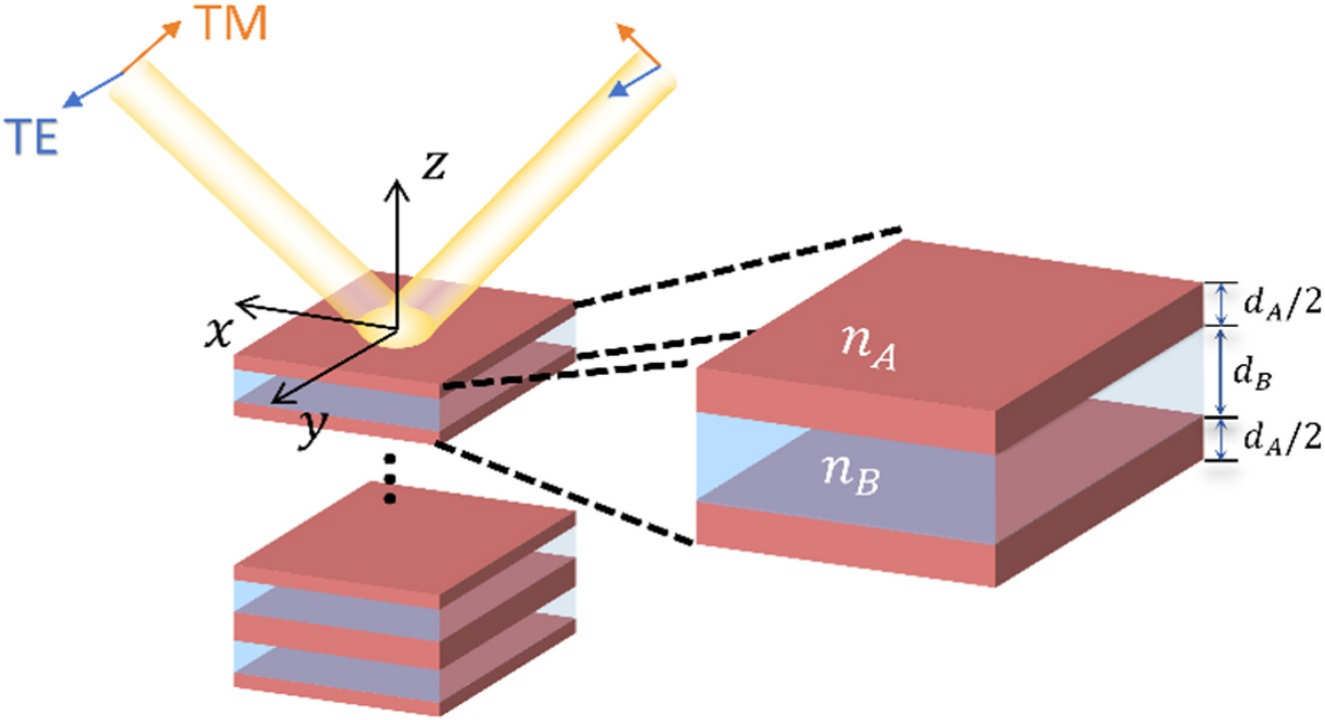
Schematic illustration of the one‐dimensional photonic crystal structure.

We derive the conditions for the emergence of band crossings for both TE and TM polarizations, and further establish the correspondence between the Brewster angle and the momentum‐space trajectory of band crossings. As a starting point, we first discuss, through numerical simulations, the band crossings that appear at different locations under TM and TE polarizations. The general condition for the occurrence of band crossings will then be derived analytically in later sections on the basis of these observations.

### 2.1 Relation Between Band Crossing and Brewster Angle

For TM‐polarized incidence, without loss of generality, we choose the structural parameters as *n*_A_ = 2, *d*_A_ = 50 nm, *n*_B_ = 1.5, and *d*_B_ = 55 nm. The calculated band structure is shown in Figure 2a. A band crossing appears near $\omega a/2c\pi =0.40,k_{y}a/2\pi =0.48$. At this point, the two bands become degenerate and exhibit linear dispersion, forming a Dirac‐cone‐like structure (see Supporting Information S1: S4). Such band crossings are of particular interest because they can serve as parent degeneracies for higher‐dimensional topological states.

**FIGURE 2:**
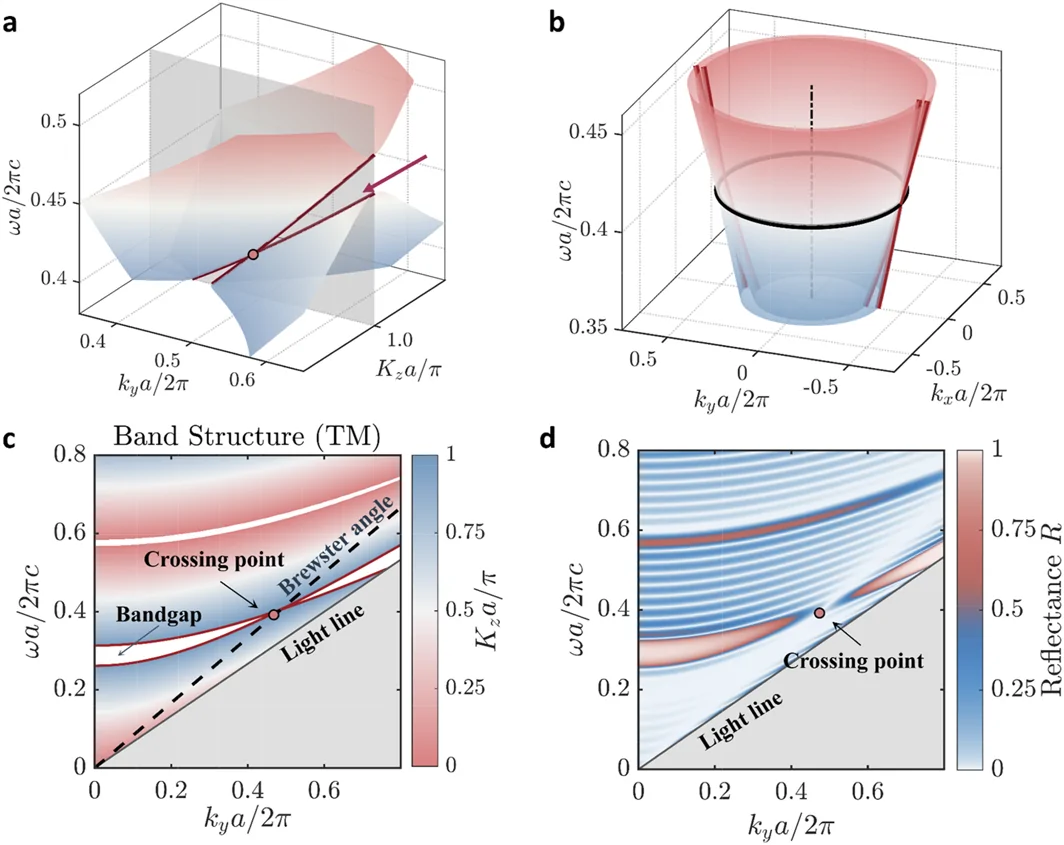
TM‐polarized band crossing associated with the Brewster condition. (a) Band structure showing a Dirac‐cone‐like crossing at the center of the Brillouin zone. (b) Extension of the band crossing (red line) into a nodal ring (black line) in three‐dimensional momentum space when the transverse momentum is included. (c) Projected band structure, where the Brewster‐condition trajectory passes through the band‐crossing point. (d) Simulated reflection spectrum of the photonic crystals with 10‐unit cells, showing a high‐transmission feature near band crossing point. In panels (c) and (d), the gray‐shaded regions indicate modes outside the momentum range accessible to external propagating waves and are therefore not directly excitable from the surrounding medium.

To illustrate this point, we further choose $K_{z}=\pi /a$, corresponding to the gray plane in Figure 2a, and include the off‐axis momentum in the perpendicular direction $k_{x}$.Both $k_{x}$ and $k_{y}$ are continuous because the structure is homogeneous along that direction. In this way, the band crossing in Figure 2a can be extended into the nodal ring shown in the three‐dimensional momentum space of Figure 2b. These effects are all rooted in band crossings in one‐dimensional topological photonics, highlighting their broader relevance to the realization of higher‐dimensional topological states that rely on band degeneracies. Therefore, clarifying the conditions under which band crossings emerge is of considerable importance not only for understanding one‐dimensional topological photonics states themselves but also for providing useful guidance for the design of band‐crossing‐enabled topological states in higher dimensions.

To better examine the condition for the occurrence of the band crossing, we plot a projected band structure in Figure 2c taken along the red arrow at the Brillouin‐zone center in Figure 2a. Remarkably, the trajectory defined by the Brewster condition at the interface between layers A and B passes exactly through the crossing point. As shown in Figure 2d, a corresponding high‐transmission region can also be observed in the reflection spectrum near the crossing point. This spectral feature is readily understood from classical optics: for TM polarization, light incident at the Brewster angle naturally gives rise to a high‐transmission line in the reflection spectrum. However, a more fundamental question is why the Brewster condition passes through the band‐crossing point, since not every high‐transmission region necessarily corresponds to a band degeneracy. As will be shown in later sections, this is not accidental, but follows directly from the general condition for band crossings derived within our theoretical framework.

To relate the momentum‐space description to experimentally controllable parameters, we consider an external coupling medium with refractive index $n_{0}=1.5$, which can be realized, for example, using a hemispherical prism. Because the transverse wave‐vector component is conserved across the interfaces, the external incidence angle $\theta_{0}$, the propagation angles $\theta_{A}$ and $\theta_{B}$ inside layers A and B, and $k_{y}$ satisfy $k_{y}=n_{0}k_{0}\;\sin\;\theta_{0}=n_{A}k_{0}\;\sin\;\theta_{A}=n_{B}k_{0}\;\sin\;\theta_{B}$, where $k_{0}=\omega /c$. For TM polarization, the Brewster condition at the A/B interface is $\tan\;\theta_{A}^{B}=n_{B}/n_{A}$, which corresponds to $k_{y}^{B}=k_{0}n_{A}n_{B}/\sqrt{n_{A}^{2}+n_{B}^{2}}$. For the parameters used in Figure 2, $n_{A}=2,n_{B}=1.5$ and $n_{0}=1.5$, we obtain $k_{y}^{B}/k_{0}=1.2$, corresponding to an external incidence angle of $\theta_{0}^{B}=53.1{}^{\circ}$. Therefore, the Brewster‐related band‐crossing trajectory shown in Figure 2 is experimentally accessible through prism coupling. The accessible momentum range for propagating excitation is determined by $\left|k_{y}\right|\leq n_{0}k_{0}$, or, in the normalized coordinates used in the figures, $\left|k_{y}a/2\pi \right|\leq n_{0}\omega a/2\pi c$. The corresponding boundary is indicated in Figure 2. The gray‐shaded region denotes states outside the experimentally accessible range, which cannot be excited by propagating waves from the external coupling medium.

### 2.2 Band Crossings Beyond the Brewster Condition

For the same structural parameters, the situation changes for TE polarization. As shown in Figure 3a,b, the band‐crossing point present for TM polarization disappears at the corresponding spectral location and is replaced by a finite band gap, which manifests itself as a high‐reflectivity region in the reflection spectrum. This behavior is expected. Under TE polarization, the Brewster effect is absent, and therefore, no persistent high‐transmission region exists at the same position. As a result, no band crossing associated with such a high‐transmission feature appears in the band structure.

**FIGURE 3:**
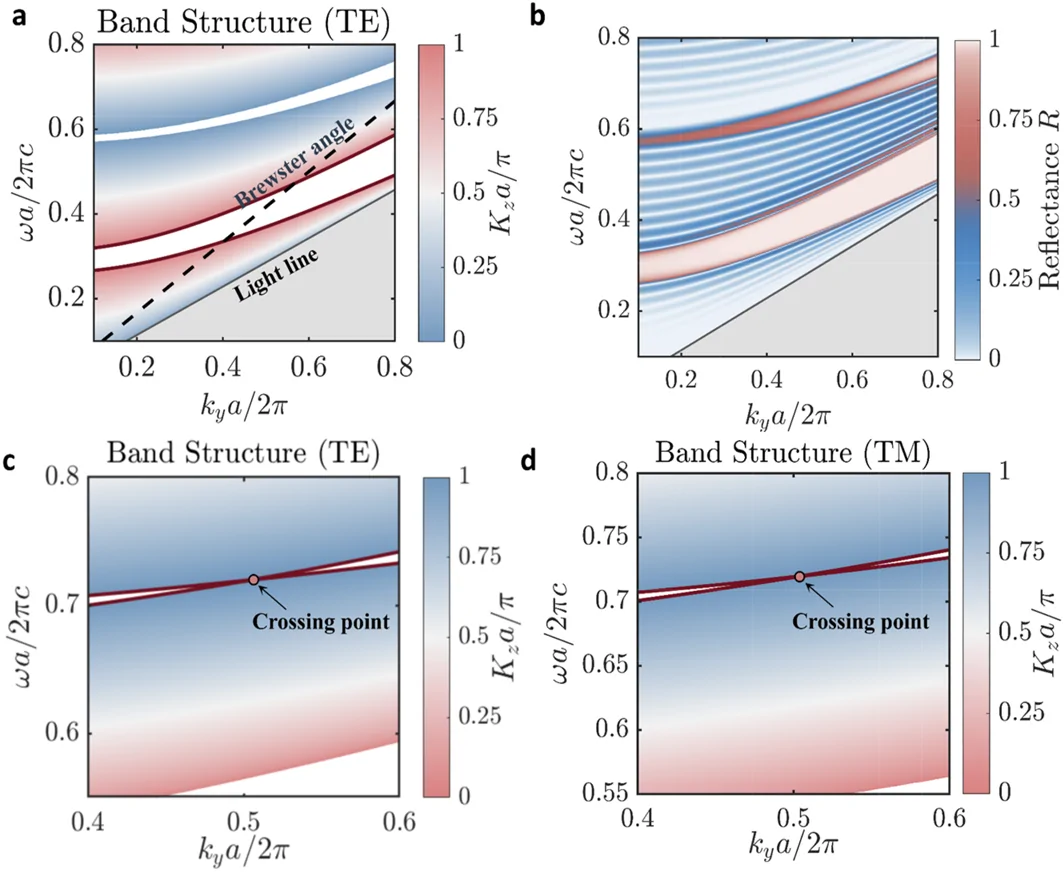
Polarization‐independent band crossings. (a) Projected band structure for TE polarization under *n*_A_ = 2, *d*_A_ = 50 nm, *n*_B_ = 1.5, and *d*_B_ = 55 nm, where the TM band crossing is replaced by a finite gap. (b) Corresponding reflection spectrum under TE polarization with the unit cell repeated ten times. The gray‐shaded regions in panels (a) and (b) denote the externally inaccessible momentum region as defined in Figure 2c,d. Projected band structures for TE and TM polarization under *n*_A_ = 3, *d*_A_ = 50 nm, *n*_B_ = 1.5, and *d*_B_ = 55 nm, showing a band crossing at the same spectral location for both polarizations. The Dirac‐cone band crossing is marked by the red line.

A natural question then arises: can band crossings still occur for TE polarization, and if so, where do they appear? To construct band crossings under TE polarization, we choose appropriate structural parameters (*n*_A_ = 3, *d*_A_ = 50 nm, *n*_B_ = 1.5, and *d*_B_ = 55 nm). Under these conditions, band crossings can also arise for TE polarization. As shown in Figure 3c, a band crossing emerges near $\omega a/2c\pi =0.72,k_{y}a/2\pi =0.50$ for TE polarization. Such a crossing does not lie on the Brewster‐angle trajectory. Moreover, unlike the polarization‐selective crossing associated with the Brewster angle, this distinctive band crossing appears at the same location for both TM and TE polarizations as shown in Figure 3d.

These observations naturally lead to a broader question: in the band structure of this system, are there more band crossings under different polarizations, and what is the general criterion that governs their occurrence? To clarify the conditions of band crossings, we analyze the dispersion relation in detail.

### 2.3 Dispersion‐Relation of the Band Crossing

In this section, we analyze the conditions for band crossing from normal incidence to oblique incidence. For simplicity, we first consider normal incidence, where the component of the incident wave vector parallel to the interface is zero ($k_{y}=0$). In this case, the TE and TM polarizations share the same dispersion relation, and it can be written in the unified form:

(1) $$\cos\left(K_{z}a\right)=\cos\left(k_{0}n_{A}d_{A}\right)\;\cos\left(k_{0}n_{B}d_{B}\right)-\frac{1}{2}\left(\frac{n_{A}}{n_{B}}+\frac{n_{B}}{n_{A}}\right)\;\sin\left(k_{0}n_{A}d_{A}\right)\sin\left(k_{0}n_{B}d_{B}\right)$$

From this relation, we obtain the following equations to describe the appearance of band‐crossing points: (see Supporting Information S1: S3)

(2) $$\left\{\begin{array}{l} \frac{1+K}{2}\cos\left(\delta_{A}+\delta_{B}\right)-\frac{K-1}{2}\cos\left(\delta_{A}-\delta_{B}\right)=\pm 1\\ -\left(A+B\right)\frac{1+K}{2}\sin\left(\delta_{A}+\delta_{B}\right)+\left(A-B\right)\frac{K-1}{2}\sin\left(\delta_{A}-\delta_{B}\right)=0 \end{array}\right.$$

where $n_{A}d_{A}/c=A$, $n_{B}d_{B}/c=B$, and $\frac{1}{2}\left(n_{A}/n_{B}+n_{B}/n_{A}\right)=K.$ Because $n_{a}\neq n_{b}$, K is a constant greater than 1. $\delta_{i}=k_{0}n_{i}d_{i}$ denotes the propagation phase accumulated by the optical wave along the z direction in layer i.

The first equation imposes a constraint on the location of band crossings, indicating that they can only appear at the high‐symmetry points of the Brillouin zone, consequently, $\cos\left(K_{z}a\right)=\pm 1$. The second equation arises from the requirement on the band variation near the band crossing. Since the band crossing occurs at a high‐symmetry point of the Brillouin zone, one has $\cos\left(K_{z}a\right)=\pm 1$. If the frequency at this point were not an extremum, then a small variation of ω would lead to $\left|\cos\left(K_{z}a\right)\right|>1$. In that case, the Bloch wave vector could no longer remain real, but would instead acquire an imaginary part. Physically, this means that no propagating Bloch state can exist in the system, and the corresponding solution becomes evanescent. The degeneracy would therefore be lifted and replaced by a band gap. Therefore, at the crossing frequency, the derivative of $\cos\left(K_{z}a\right)$ with respect to ω must vanish.

From the above relations, we obtain the phase criterion:

$$\delta_{A}=m\pi ,\delta_{B}=n\pi$$

Here, m,n are both positive integers. This result indicates that band crossings occur only when the propagation phases accumulated in both layers are integer multiples of π. As we will demonstrate below, this phase criterion also remains valid under oblique incidence and therefore provides a general criterion for determining when band crossings occur in one‐dimensional topological photonics.

We next consider oblique incidence, where the incident wave vector has a finite in‐plane component. In this case, the dispersion relation becomes polarization‐dependent, and the TE and TM modes must be treated separately.

For TE polarization:

(3) $$\cos\left(K_{z}a\right)=\cos\left(k_{zA}d_{A}\right)\;\cos\left(k_{zB}d_{B}\right)-\frac{1}{2}\left(\frac{\mu_{B}k_{zA}}{\mu_{A}k_{zB}}+\frac{\mu_{A}k_{zB}}{\mu_{B}k_{zA}}\right)\;\sin\left(k_{zA}d_{A}\right)\sin\left(k_{zB}d_{B}\right)$$

For TM polarization:

(4) $$\cos\left(K_{z}a\right)=\cos\left(k_{zA}d_{A}\right)\;\cos\left(k_{zB}d_{B}\right)-\frac{1}{2}\left(\frac{\varepsilon_{B}k_{zA}}{\varepsilon_{A}k_{zB}}+\frac{\varepsilon_{A}k_{zB}}{\varepsilon_{B}k_{zA}}\right)\;\sin\left(k_{zA}d_{A}\right)\sin\left(k_{zB}d_{B}\right)$$

w here $k_{zi}=\sqrt{k_{0}^{2}\varepsilon_{i}-k_{y}^{2}}=k_{0}\sqrt{\varepsilon_{i}-k_{y}^{2}/k_{0}^{2}}=k_{0}\tilde{n_{i}}$.

For simplicity, we further define $\tilde{n_{A}}d_{A}/c=A$, $\tilde{n_{B}}d_{B}/c=B$ and $\frac{1}{2}\left(\frac{\mu_{B}k_{zA}}{\mu_{A}k_{zB}}+\frac{\mu_{A}k_{zB}}{\mu_{B}k_{zA}}\right)=K\left(TE\right)$, $\frac{1}{2}\left(\frac{\varepsilon_{B}k_{zA}}{\varepsilon_{A}k_{zB}}+\frac{\varepsilon_{A}k_{zB}}{\varepsilon_{B}k_{zA}}\right)=K\left(TM\right)$. We can again obtain

(5) $$\left\{\begin{array}{l} \frac{1+K}{2}\cos\left(\delta_{A}+\delta_{B}\right)-\frac{K-1}{2}\cos\left(\delta_{A}-\delta_{B}\right)=\pm 1\\ -\left(A+B\right)\frac{1+K}{2}\sin\left(\delta_{A}+\delta_{B}\right)+\left(A-B\right)\frac{K-1}{2}\sin\left(\delta_{A}-\delta_{B}\right)=0 \end{array}\right.$$

When K>1, Equations (2) and (5) are only different in coefficient, and the solution is the same. It follows in the same way that when $\delta_{A}=m\pi$ and $\delta_{B}=n\pi$, band crossing appears. The crossing point is located in the high‐symmetry region of the Brillouin zone, where $\cos\left(K_{z}a\right)=\pm 1$. In other words, under oblique incidence, band crossing still occurs when the propagation phase of light in layers A and B is an integer multiple of π for both TM and TE polarizations.

We further verify our conclusion numerically. Taking the same structural parameters used in Figure 3c,d (*n*_A_ = 3, *d*_A_ = 50 nm, *n*_B_ = 1.5, and *d*_B_ = 55 nm) as an example, we focus on TE polarization; the same phenomenon also appears in the TM polarization. As shown in Figure 4a,b, a band crossing appears near ωa/2cπ=0.72 and $k_{y}a/2\pi =0.50$. In Figure 4c, around the band‐crossing point of conical high‐reflection region, a high‐transmission area can be clearly observed. At the same time, Figure 4d shows that the phase accumulations in the layers are exactly integer multiples of π. As shown in Figure 4e,f, when the frequency range under consideration is extended, the numerical results remain in excellent agreement with the theoretical prediction: band crossings appear whenever the propagation phases in the layers are integer multiples of π. For the band crossing shown in Figure 3c,d, the accumulated phase along the z direction in layer A is $\delta_{A}=2\pi$, and that in layer B is $\delta_{B}=\pi$, in agreement with our theory.

**FIGURE 4:**
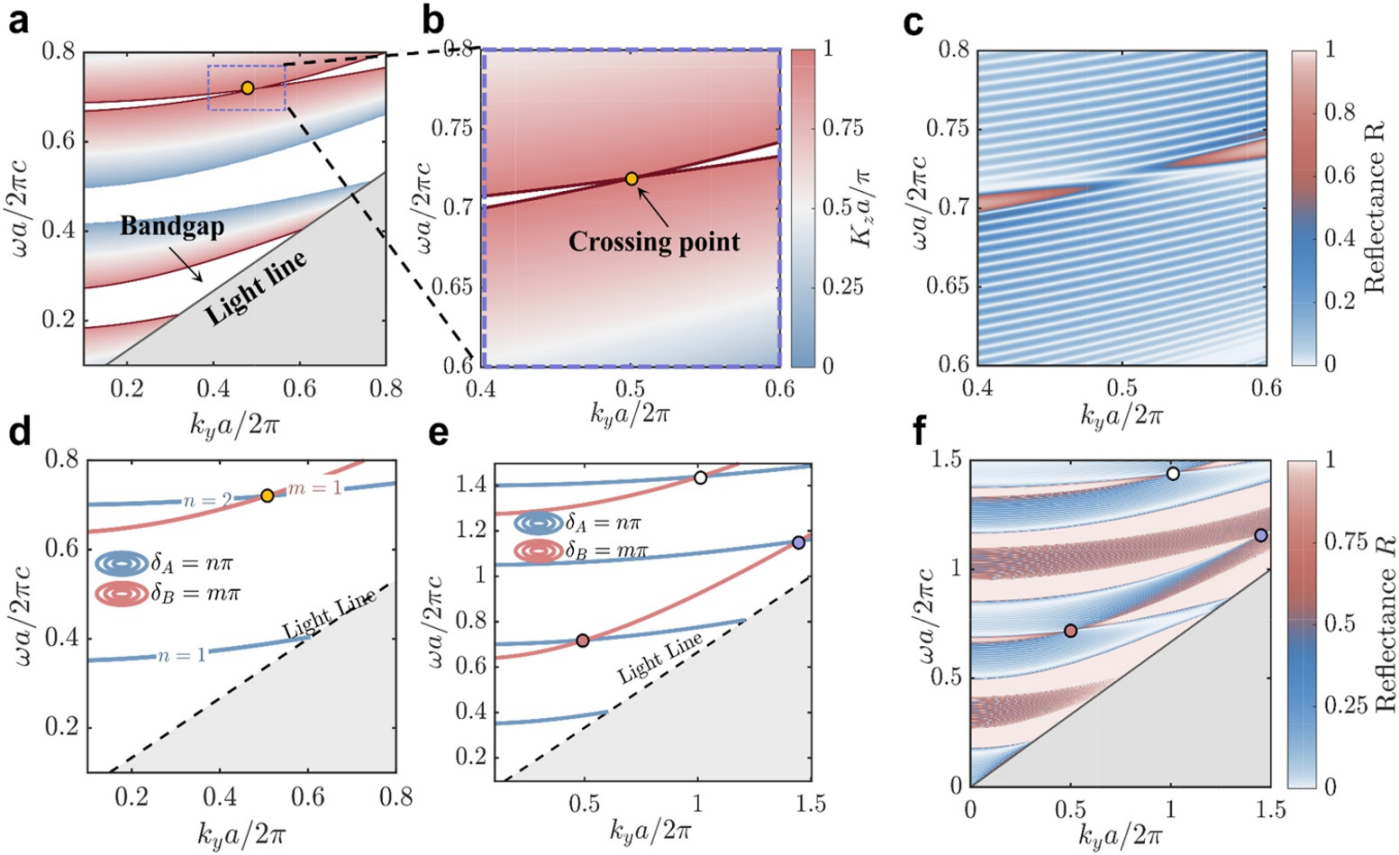
Numerical verification of the unified phase criterion for TE polarization. (a) Projected band structure for TE polarization. (b) Enlarged view of the boxed region in panel (a), showing the Dirac‐cone‐like band crossing. (c) Reflection spectrum around the boxed region in panel (a), showing the conical high‐reflection region. (d) Phase accumulation in the layers, showing that band crossing appears exactly when $\delta_{A}=2\pi$ and $\delta_{B}=\pi$. (e) Phase map over a broader frequency range, with the predicted crossing points marked by dots where accumulated layer phases are all integer multiples of π. (f) Mapping of reflection spectrum corresponding to (e). The gray‐shaded regions have the same meaning as in Figure 2 and indicate the externally inaccessible momentum region.

### 2.4 Brewster Angle as a Trajectory of Band Degeneracies

The above derivation was carried out for the general case of K>1. We now turn to a special case of Equation (5), namely K=1. If K=1, then Equation (5) becomes

(6) $$\begin{array}{l} \cos\left(\delta_{A}+\delta_{B}\right)=\pm 1\\ \sin\left(\;\delta_{A}+\delta_{B}\right)=0 \end{array}$$

Under this simplification, the crossing condition reduces to $\delta_{A}+\delta_{B}=m\pi$, which means that band crossings occur whenever the total propagation phase accumulated across layers A and B is an integer multiple of π. As the incident frequency increases, this condition is satisfied repeatedly, giving rise to a sequence of band crossings.

Moreover, K=1 implies: $k_{za}\varepsilon_{b}=k_{zb}\varepsilon_{a}$, from which one further obtains:

(7) $$\frac{k_{0}^{2}\varepsilon_{a}-k_{y}^{2}}{k_{0}^{2}\varepsilon_{a}}=\frac{k_{y}^{2}}{k_{0}^{2}\varepsilon_{b}}$$

That is:

(8) $$\cos\left(\theta_{a}\right)=\sin\left(\theta_{b}\right)$$

This indicates that the incident wave propagates along the Brewster‐angle direction at the A/B interface. Therefore, under Brewster‐angle incidence, discrete degenerate frequencies $\omega_{m}$ emerge in the Brillouin‐zone, forming a series of band crossings. That is, when the band structure reaches the Brillouin‐zone boundary or center along the Brewster‐angle direction, the gap closes and band crossings emerge naturally. From this viewpoint, the Brewster angle can be understood as a collection of band‐crossing points.

To illustrate this point more clearly, we further consider disordered structures. Since the Brewster angle depends only on the refractive indices of the two media and is independent of their thicknesses, one may expect that, regardless of how the unit‐cell geometry is modified, band‐crossing points associated with the Brewster‐angle direction will still appear in momentum space, with only their positions shifted. This further implies that, if sufficiently many unit cells with the same refractive indices but different thicknesses are stacked together, their band‐crossing points will become densely distributed along the Brewster‐related line in momentum space. Correspondingly, the high‐transmission features induced by these crossings will merge into a line‐like transmission region in the reflection spectrum. However, away from this line, disorder makes the high‐transmission features of different unit cells no longer aligned, so they are gradually smeared out.

To verify this physical scenario statistically, we further consider disordered multilayer structures. As shown schematically in Figure 5a, we stack 400 copies of the original unit cell and independently perturb the thickness of each layer, whereas keeping the refractive indices unchanged. Three disorder strengths of 0%, 10%, and 100% are considered. For each disorder strength, 20 independent random realizations are generated and the corresponding reflectance maps are ensemble averaged.

**FIGURE 5:**
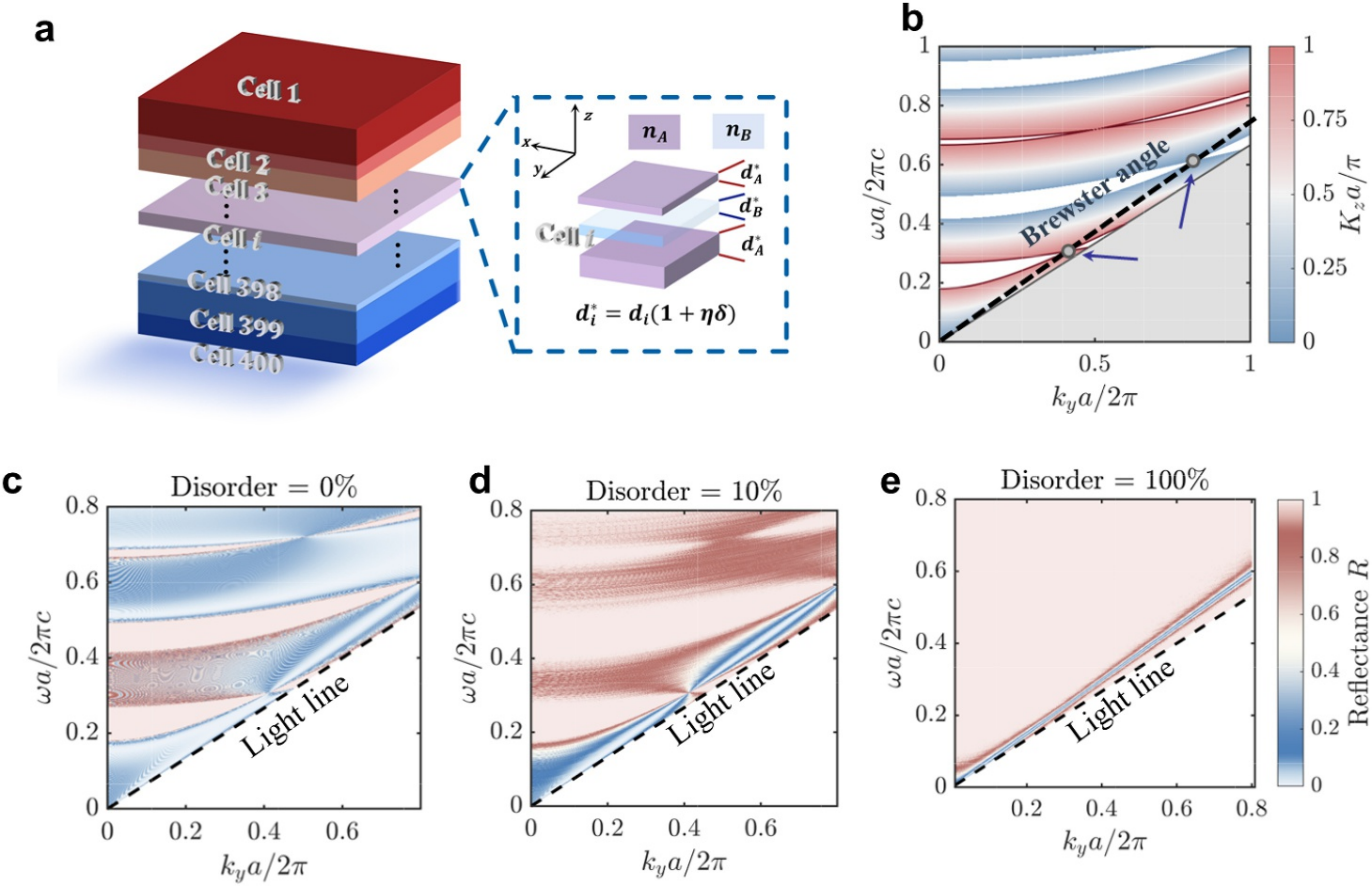
Brewster‐angle‐related transmission channel in disordered multilayers. (a) Schematic of the structure after introducing thickness disorder. (b) Band structure for TM polarization with $n_{A}=3$, $d_{A}=25$ nm, $n_{B}=1.5$, and $d_{B}=55$ nm; the band crossings on the Brewster‐angle line are indicated by the purple arrows. (c)–(e) Ensemble‐averaged reflectance maps for 0%, 10%, and 100% thickness disorder, respectively, each averaged over 20 independent disorder realizations. Increasing disorder suppresses the background interference, whereas the Brewster‐angle‐related high‐transmission channel remains clearly preserved.

Since the Brewster condition depends only on the refractive‐index contrast at the interface and is independent of the layer thicknesses, thickness disorder changes the accumulated propagation phases but does not shift the Brewster‐angle trajectory itself. Away from this trajectory, the random thickness variations progressively decorrelate the interference conditions among different realizations. Consequently, as shown in Figure 5c–5e, the ensemble averaging suppresses realization‐dependent background interference, whereas the high‐transmission channel remains clearly aligned with the Brewster direction even for 100% thickness disorder. These results demonstrate that the observed transmission channel is not associated with a particular random configuration but is robust against the considered thickness disorder. Further realization‐resolved statistical analysis is provided in Supporting Information S1: Section S7.

## 3 Conclusion

In summary, we have established a unified phase criterion for band crossings in one‐dimensional photonic crystals and shown that these degeneracies arise when the propagation phases accumulated in both constituent layers are integer multiples of π. In classical optics, multilayer dielectric structures similar to one‐dimensional photonic crystals are widely used in Bragg mirrors, chirped mirrors, high‐reflection coatings, and other optical components [42, 43, 44, 45, 46], where accumulated phase plays a central role in determining the reflection and transmission properties. From the viewpoint of band theory, our results show that band crossings in one‐dimensional topological photonics are governed by the same factor, namely, the phase accumulation in the constituent layers. This unified phase criterion applies to both TE and TM polarizations and remains valid under oblique incidence. We also explain why the crossings appear at high‐symmetry regions of the Brillouin zone and exhibit Dirac‐cone dispersions. (See Supporting Information S1: S4).

Beyond clarifying the band‐crossing formation mechanism, our results also strengthen the connection between classical optics and topological photonics. In particular, we demonstrated that the Brewster angle for TM polarization can be reinterpreted as an angle‐dependent trajectory of band degeneracies, linking a familiar interface‐optics effect to the emergence of topological phase transition points in periodic media. As a result, the corresponding crossing features may appear in the reflection spectrum as a clear line‐like transmission region, which could be useful for realizing angle‐selective optical filters and related photonic devices [47, 48]. Moreover, by translating the unified phase criterion into device‐relevant design rules, this framework may help improve existing multilayer photonic devices. The disorder‐robust Brewster‐angle‐related transmission line suggests enhanced tolerance and stability in angle‐selective dielectric coatings [41], whereas the polarization‐independent band crossings provide a promising route toward polarization‐insensitive one‐dimensional topological photonic devices and polarization‐independent extensions of nodal‐line or nodal‐ring‐like degeneracies [35]. More broadly, the unified phase picture developed here provides a practical framework for engineering polarization‐ and angle‐dependent band crossings, and may serve as a useful guide for designing layered photonic structures with tailored dispersion, selective transmission, and band‐degeneracy‐enabled topological functionalities.

## Author Contributions

**Jingxuan Song:** writing – original draft, writing – review and editing, data curation, formal analysis, investigation, validation, visualization, software. **Haiyi Liu:** writing – review and editing, validation, formal analysis, investigation. **Changlin Sun:** writing – review and editing, validation, formal analysis, investigation. **Pengfei Qi:** writing – original draft, writing – review and editing, conceptualization, methodology, funding acquisition, project administration, investigation. **Weiwei Liu:** writing – review and editing, funding acquisition.

## Funding

This work was supported by the National Natural Science Foundation of China (Grants 12574418 and 12104241), Natural Science Foundation of Tianjin (Grant 25JCYBJC01310), and Fundamental Research Funds for the Central Universities (Grants 075‐63263241, 075‐9261500147, 075–63263235, and 075‐63261015).

## Conflicts of Interest

The authors declare no conflicts of interest.

## Supporting information


Supporting Information S1

## Data Availability

Data underlying the results presented in this paper are not publicly available at this time but may be obtained from the authors upon reasonable request.
