# Supplementary material for: Unified Phase Criterion for Band Crossings in One‐Dimensional Topological Photonics
Source: Nanophotonics. 2026 Nov 6;15(21):e70329. doi: 10.1002/nap2.70329 (PMC13743866; doi:10.1002/nap2.70329)
Supplement: Supplementary file 1 [file NAP2-15-e70329-s001.docx]

supplemental document

1. Transfer Matrix Method

As illustrated in Fig. S1(a), the proposed structure is a one-dimensional periodic crystal composed of repeating unit cells. We can model the propagation of light through this structure as a series of transmissions across consecutive interfaces. By applying the electromagnetic boundary conditions derived from Maxwell's equations, we can establish the relationship between the optical fields at these interfaces [1].

Specifically, we first consider a single-layer structure as shown in Fig. S1(b). Let $E_{0}$ and $H_{0}$ denote the field vectors on the $n_{0}$ side of interface Ⅰ, while $E_{1}$ and $H_{1}$ denote the field vectors on the $n_{1}$ side of the interface Ⅱ. At interface Ⅰ, the optical fields consist of the incident wave $E_{i1}$, the transmitted wave $E_{t1}$, the reflected wave $E_{r1}$, and the reflected wave $E_{r2}^{`}$returning from interface Ⅱ.

Fig. S1 Schematic diagram of structure and interface

Assuming the medium is free of charges and conduction currents, and applying the electromagnetic boundary conditions for the TE mode, we obtain the following equations:

|  | $\left\{\begin{matrix} E_{i1}+E_{r1}=E_{t1}+E_{r2}^{'} \\ H_{i1}cos\left(\theta_{0}\right)-H_{r1}cos\left(\theta_{0}\right)=H_{t1}cos\left(\theta_{1}\right)-H_{r2}^{'}cos\left(\theta_{1}\right) \end{matrix}\right.$ | (S1) |
| --- | --- | --- |

Additionally, the phase evolution between the interfaces gives:

|  | $\left\{\begin{matrix} E_{i2}=E_{t1}e^{i\delta} \\ E_{r2}^{'}=E_{r2}e^{-i\delta} \end{matrix}\right.$ | (S2) |
| --- | --- | --- |

Where:

$$\delta=k_{z}d$$

For the monochromatic plane waves,we have:

$$H=\sqrt{\frac{\varepsilon}{\mu}}E=\sqrt{\frac{\varepsilon_{0}}{\mu_{0}}}E\sqrt{\varepsilon_{r}}$$

From the above relations, we can derive the fields at Interface II in terms of Interface Ⅰ:

|  | $\left\{\begin{matrix} E_{1}=cos\left(\delta\right)E_{2}-\frac{i}{\eta_{1}}sin\left(\delta\right)H_{2} \\ H_{1}=-i\eta_{1}sin\left(\delta\right)E_{2}+cos\left(\delta\right)H_{2} \end{matrix}\right.$ | (S3) |
| --- | --- | --- |

where $\eta_{1}$ is the effective impedance of the medium.

$$\eta_{1}=\sqrt{\frac{\varepsilon_{0}}{\mu_{0}}}\sqrt{\varepsilon_{1}}cos^{2}\left(\theta_{1}\right)$$

Expressed in matrix form , this becomes:

|  | $\left[\begin{matrix} E_{1} \\ H_{1} \end{matrix}\right]=\left[\begin{matrix} cos\left(\delta\right) & -\frac{i}{\eta_{1}}sin\left(\delta\right) \\ -i\eta_{1}sin\left(\delta\right) & cos\left(\delta\right) \end{matrix}\right]\left[\begin{matrix} E_{2} \\ H_{2} \end{matrix}\right]$ | (S4) |
| --- | --- | --- |

A similar result can be obtained for TM polarization, with the corresponding effective impedance $\eta_{1}$. Thus, we obtain the transfer matrix $M_{01}$ for a single dielectric layer:

|  | $M_{01}=\left[\begin{matrix} cos\left(\delta\right) & -\frac{i}{\eta_{1}}sin\left(\delta\right) \\ -i\eta_{1}sin\left(\delta\right) & cos\left(\delta\right) \end{matrix}\right]$ | (S5) |
| --- | --- | --- |

For the periodic structure shown in Fig. S1(a), this transfer matrix can be applied sequentially layer by layer:

|  | $\left[\begin{matrix} E_{1} \\ H_{1} \end{matrix}\right]=M_{01}M_{12}M_{23}...M_{N-1N}\left[\begin{matrix} E_{N} \\ H_{N} \end{matrix}\right]$ | (S6) |
| --- | --- | --- |

This matrix method can be utilized to calculate the dispersion relations, reflectance, and transmittance of the material.

2. Derivation of Dispersion Relations using Bloch's Theorem

For the structure shown in Fig. S1(a), when the number of periods N is sufficiently large, it can be approximated as an infinite periodic structure. According to Bloch's theorem, the field vectors at the boundaries of a unit cell satisfy:

|  | $\left[\begin{matrix} E_{N-1} \\ H_{N-1} \end{matrix}\right]=e^{-iqa}\left[\begin{matrix} E_{N} \\ H_{N} \end{matrix}\right]$ | (S7) |
| --- | --- | --- |

where $q$ is the Bloch wave vector and $a$ is the lattice period.

Simultaneously, based on the transfer matrix method, we have:

|  | $\left[\begin{matrix} E_{N-1} \\ H_{N-1} \end{matrix}\right]=M_{unit}\left[\begin{matrix} E_{N} \\ H_{N} \end{matrix}\right]$ | (S8) |
| --- | --- | --- |

Equating the two expressions yields an eigenvalue equation:

|  | $M_{unit}\left[\begin{matrix} E_{N} \\ H_{N} \end{matrix}\right]=e^{-iqa}\left[\begin{matrix} E_{N} \\ H_{N} \end{matrix}\right]$ | (S9) |
| --- | --- | --- |

By imposing the condition for non-trivial solutions, $\text{det}\left(M_{cell}-e^{-iKa}\right)=0$,we obtain the dispersion relations for the one-dimensional periodic structure.

For TE polarization:

|  | $cos\left(K_{z}a\right)=cos\left(k_{zA}d_{A}\right)cos\left(k_{zB}d_{B}\right)-\frac{1}{2}\left(\frac{\mu_{B}k_{zA}}{\mu_{A}k_{zB}}+\frac{\mu_{A}k_{zB}}{\mu_{B}k_{zA}}\right)sin\left(k_{zA}d_{A}\right)sin\left(k_{zB}d_{B}\right)$ | (S10) |
| --- | --- | --- |

For TM polarization:

|  | $cos\left(K_{z}a\right)=cos\left(k_{zA}d_{A}\right)cos\left(k_{zB}d_{B}\right)-\frac{1}{2}\left(\frac{\varepsilon_{B}k_{zA}}{\varepsilon_{A}k_{zB}}+\frac{\varepsilon_{A}k_{zB}}{\varepsilon_{B}k_{zA}}\right)sin\left(k_{zA}d_{A}\right)sin\left(k_{zB}d_{B}\right)$ | (S11) |
| --- | --- | --- |

3. Analysis of the Dispersion Relations

3.1 Dispersion and Band Relations under Normal Incidence

In this section, we first examine the case of normal incidence, where the in-plane component of the incident wave vector is zero .$\left(k_{y}=0\right)$.. Under this condition, the TE and TM modes share an identical dispersion relation. The band structure of the 1D binary photonic crystal can thus be described by a unified equation:

|  | $cos\left(K_{z}a\right)=cos\left(k_{0}n_{A}d_{A}\right)cos\left(k_{0}n_{B}d_{B}\right)-\frac{1}{2}\left(\frac{n_{A}}{n_{B}}+\frac{n_{B}}{n_{A}}\right)sin\left(k_{0}n_{A}d_{A}\right)sin\left(k_{0}n_{B}d_{B}\right)$ | (S12) |
| --- | --- | --- |

Note that the right side of the equation depends solely on the incident light frequency $\text{ω}$. We define the right side as $F\left(\text{ω}\right)$,a function determined entirely by the propagation phases in the two dielectric layers and their material parameters.

3.2 Crossings Are Restricted to High-Symmetry Points of the Brillouin Zone

To understand the spatial constraints on band crossings, we investigate the behavior of the dispersion relation $cos\left(K_{z}a\right)=F\left(\text{ω}\right)$ near a band crossing point.

For a given Bloch wave vector$q$, the term$cos\left(K_{z}d\right)$ is a constant, while $F\left(\text{ω}\right)$ is a continuous function of $\text{ω}$.

Near a band crossing, a single Bloch wave vector must correspond to two distinct eigenfrequencies, meaning a specific value of $cos\left(K_{z}a\right)$ maps to two different $\text{ω }$values.

This implies that the function $F\left(\text{ω}\right)$ must be non-monotonic near this frequency; it must exhibit an extremum, allowing one function value to correspond to two independent variables locally.

However, within the Brillouin zone, $cos\left(K_{z}a\right)$only reaches its extrema at the high-symmetry points where $cos\left(K_{z}a\right)=\pm1$. Therefore, the dispersion relation can only permit one $cos\left(K_{z}a\right)$ to correspond to two frequency solutions locally if the band crossing occurs at these symmetry points. This leads to an important conclusion: under normal incidence, band crossings in 1D photonic crystals can only occur at high-symmetry points of the Brillouin zone, namely at the zone center or boundaries.

3.3 Phase Conditions for Band Crossings

Building upon the previous section, we established that under normal incidence, band crossings can only occur where $cos\left(K_{z}a\right)=\pm1$. In this section, we further derive the phase conditions required for these crossings to manifest.

For normal incidence, the unified dispersion relation can be rewritten as:

|  | $cos\left(K_{z}a\right)=cos\left(\delta_{A}\right)cos\left(\delta_{B}\right)-Ksin\left(\delta_{A}\right)sin\left(\delta_{B}\right)$ | (S13) |
| --- | --- | --- |

Where:

$$\delta_{A}=k_{0}n_{A}d_{A},\delta_{B}=k_{0}n_{B}d_{B},K=\frac{1}{2}\left(\frac{n_{b}}{n_{a}}+\frac{n_{a}}{n_{b}}\right)$$

As established in Sec. 3.2, a band crossing under normal incidence can occur only at a high-symmetry point of the Brillouin zone, where $\cos\left( qa \right)=\pm1$. To determine when the bandgap closes at such a$cos\left(K_{z}a\right)=cos\left(\delta_{A}\right)cos\left(\delta_{B}\right)-Ksin\left(\delta_{A}\right)sin\left(\delta_{B}\right)$ point, we write the right-hand side of Eq. (S13) as $F\left( \omega\right)$, such that

$\cos(K_{z}a)=F(\omega)$.

For propagating Bloch modes, the allowed spectral region satisfies$\left|F(\omega)\right|\leq1$ , whereas corresponds to a bandgap. Therefore, a band crossing corresponds to the situation in which $F(\omega)$touches the boundary $+1$or $-1$at an extremum, rather than passing beyond it into the bandgap. Consequently, at the crossing frequency$\omega_{0}$, the following two conditions must be satisfied simultaneously

(1)
(2)

Based on this relationship, we can derive the following equation:

|  | $\left\{\begin{matrix} \frac{1+K}{2}\cos(\delta_{A}+\delta_{B})-\frac{K-1}{2}\cos(\delta_{A}-\delta_{B})=\pm1 \\ -\left(A+B\right)\frac{1+K}{2}\sin(\delta_{A}+\delta_{B})+\left(A-B\right)\frac{K-1}{2}\sin\left(\delta_{A}-\delta_{B}\right)=0 \end{matrix}\right.$ | (S14) |
| --- | --- | --- |

where:

$$\begin{matrix} \frac{n_{A}d_{A}}{c}=A\text{，}\frac{n_{B}d_{B}}{c}=B\text{，} \\ \frac{1}{2}\left(\frac{n_{A}}{n_{B}}+\frac{n_{B}}{n_{A}}\right)=K. \end{matrix}$$

Equation (S14) contains only the sine and cosine functions of the phase sum $\delta_{A}+\delta_{B}$and phase difference $\delta_{A}-\delta_{B}$. To simplify the algebra without introducing any additional physical assumptions, we define the following auxiliary variables:

|  | $\left\{\begin{matrix} \cos(\delta_{A}+\delta_{B})=x_{1},\cos(\delta_{A}-\delta_{B})=x_{2} \\ \sin(\delta_{A}+\delta_{B})=y_{1},\sin\left(\delta_{A}-\delta_{B}\right)=y_{2} \end{matrix}\right.$ | (S15) |
| --- | --- | --- |

Here,$x_{1}$and $y_{1}$represent the cosine and sine of the phase sum, whereas $x_{2}$ and $y_{2}$represent those of the phase difference. Therefore, these variables are not independent; each pair must satisfy the trigonometric identity $x_{i}^{2}+y_{i}^{2}=1$.

Using these definitions, Eq. (S14), together with the two trigonometric identities, can be rewritten as Eq. (S16):

|  | $\left\{\begin{matrix} x_{1}^{2}+y_{1}^{2}=1 \\ x_{2}^{2}+y_{2}^{2}=1 \\ \frac{1+K}{2}x_{1}-\frac{K-1}{2}x_{2}=\pm1 \\ -(A+B)\frac{1+K}{2}y_{1}+(A-B)\frac{K-1}{2}y_{2}=0 \end{matrix}\right.$ | (S16) |
| --- | --- | --- |

We next eliminate $x_{1}$and$y_{1}$, because the final phase condition can be determined from the phase-difference variables $x_{2}$and$y_{2}$. Specifically, the third and fourth equations in Eq. (S16) are first solved for $x_{1}$and$y_{1}$, respectively. Substituting these expressions into the first constraint, $x_{1}^{2}+y_{1}^{2}=1$, while retaining$x_{2}^{2}+y_{2}^{2}=1$, gives Eq. (S17).

|  | $\left\{\begin{matrix} \left[(\frac{K-1}{2}x_{2}\pm1)\frac{2}{K+1}\right]^{2}+\left[\frac{(A-B)(K-1)}{(A+B)(K+1)}y_{2}\right]^{2}=1 \\ x_{2}^{2}+y_{2}^{2}=1 \end{matrix}\right.$ | (S17) |
| --- | --- | --- |

that is:

|  | $\left[(\frac{K-1}{2}x_{2}\pm1)\frac{2}{K+1}\right]^{2}+\left[\frac{(A-B)(K-1)}{(A+B)(K+1)}\right]^{2}(1-x_{2}^{2})=1$ | (S18) |
| --- | --- | --- |

For the equation:

|  | $\left[(\frac{K-1}{2}x_{2}+1)\frac{2}{K+1}\right]^{2}+\left[\frac{(A-B)(K-1)}{(A+B)(K+1)}\right]^{2}(1-x_{2}^{2})=1$ | (S19) |
| --- | --- | --- |

Equation (S18) contains the two possible high-symmetry conditions corresponding to$\cos(K_{z}a)=+1$ and$\cos(K_{z}a)=-1$. We therefore consider these two branches separately. An important constraint is that$-1\leq x_{2}=\cos(\delta_{A}-\delta_{B})\leq+1$, so only algebraic solutions lying within this interval are physically admissible.

For the $+1$branch, Eq. (S18) reduces to Eq. (S19). Solving this equation gives $x_{2}=1$as the physically admissible solution. The other algebraic solution lies outside the allowed interval $[-1,1]$for the cosine function and must therefore be discarded. Substituting $x_{2}=1$into Eq. (S16) then gives$x_{1}=1$.

Therefore,

$$\cos(\delta_{A}+\delta_{B})=1,\cos(\delta_{A}-\delta_{B})=1$$

which gives

$$\delta_{A}+\delta_{B}=2q\pi,\delta_{A}-\delta_{B}=2p\pi$$

where $p$and $q$are integers.

For the $-1$branch, Eq. (S18) becomes Eq. (S20).

|  | $\left[(\frac{K-1}{2}x_{2}-1)\frac{2}{K+1}\right]^{2}+\left[\frac{(A-B)(K-1)}{(A+B)(K+1)}\right]^{2}(1-x_{2}^{2})=1$ | (S20) |
| --- | --- | --- |

In this case, the physically admissible solution is$x_{2}=-1$,Substituting $x_{2}=-1$into Eq. (S16) gives$x_{1}=-1$. Hence:

$$\cos(\delta_{A}+\delta_{B})=-1,\cos(\delta_{A}-\delta_{B})=-1$$

leading to:

$$\delta_{A}+\delta_{B}=(2q+1)\pi,\delta_{A}-\delta_{B}=(2p+1)\pi$$

In both branches, the sum and difference of the two layer phases are integer multiples of $\pi$with the same parity. Since:

$$\delta_{A}=\frac{(\delta_{A}+\delta_{B})+(\delta_{A}-\delta_{B})}{2},\delta_{B}=\frac{(\delta_{A}+\delta_{B})-(\delta_{A}-\delta_{B})}{2}$$

the phase accumulated in each individual layer must itself be an integer multiple of $\pi$. Therefore, both branches lead to the same compact phase condition:

$$\delta_{A}=m\pi,\delta_{B}=n\pi$$

Here, $m,n$ are all positive integers.

As illustrated in Fig. S2, band crossings will only emerge when these phase conditions are satisfied.

Fig. S2. Interlayer phase distribution and band crossings under normal incidence. (a) Phase accumulation of optical waves in layers A and B under normal incidence.(b) The band structure under normal incidence with *n_A_* = 3, *d_A_* = 25 nm, *n_B_* = 1.5, *d_B_* = 55 nm.

3.4 Generalization to Oblique Incidence

We now extend our analysis to the case of oblique incidence, where the incident wave vector possesses a finite in-plane component. Because the dispersion relation becomes polarization-dependent, the TE and TM modes must be treated separately.

By defining the generalized phase accumulations :

$$k_{zA}d_{A}=\delta_{A}\text{，}k_{zB}d_{B}=\delta_{B}\text{，}\frac{1}{2}\left(\frac{\mu_{B}k_{zA}}{\mu_{A}k_{zB}}+\frac{\mu_{A}k_{zB}}{\mu_{B}k_{zA}}\right)=K\left(TM\right),\frac{1}{2}\left(\frac{\varepsilon_{B}k_{zA}}{\varepsilon_{A}k_{zB}}+\frac{\varepsilon_{A}k_{zB}}{\varepsilon_{B}k_{zA}}\right)=K\left(TE\right)$$

where:

$$k_{zi}=\sqrt{k_{0}^{2}\varepsilon_{i}-k_{y}^{2}}=k_{0}\sqrt{\varepsilon_{i}-\frac{k_{y}^{2}}{k_{0}^{2}}}=k_{0}\widetilde{n_{i}}$$

we can reduce both equations back to the unified form:

|  | $cos\left(K_{z}a\right)=cos\left(\delta_{A}\right)cos\left(\delta_{B}\right)-Ksin\left(\delta_{A}\right)sin\left(\delta_{B}\right)$ | (S21) |
| --- | --- | --- |

Following the same logic as the normal incidence case, we derive identical mathematical requirements. Thus, under oblique incidence, band crossings still occur strictly when $\delta_{A}=m\pi$ and $\delta_{B}=n\pi$, and these crossing points remain localized at the high-symmetry regions of the Brillouin zone where $cos\left(qa\right)=\pm1$.

4. Proof of Linear Dispersion

We take the dispersion relation under normal incidence as an example; a similar proof holds for oblique incidence [2]. At the band crossing point$(\omega_{0},K_{z0})$, we have:

$$cos\left(K_{z0}d\right)=\pm1,k_{0}n_{A}d_{A}=n\pi,k_{0}n_{B}d_{B}=m\pi$$

let $(\omega_{1},K_{z1})$ be a point in the immediate vicinity of $(\omega_{0},K_{z0})$. By expanding the dispersion relation to the lowest non-vanishing order for small perturbations, we obtain:

|  |  | (S22) |
| --- | --- | --- |

Consequently:

|  | $\left\vert q_{1}-q_{0}\left\vert\propto\right\vert\omega_{1}-\omega_{0}\right\vert$ | (S23) |
| --- | --- | --- |

This clearly demonstrates that the dispersion relation is linear in the vicinity of the band crossing point, characteristic of a Dirac cone.

5. Band Crossings under TM Polarization

Fig. S3. Interlayer phase distribution and band crossings under TM Polarization. (a)(b) The band structure and its magnified view under TM polarization. Compared to TE polarization, additional crossing points emerge along the Brewster angle trajectory. (c) Reflectance profile near the band crossing points under TM polarization. (d)(e) Phase accumulation diagrams for the optical waves in the layers. (f) Reflectance profile over an extended frequency range, revealing newly formed transmission regions along the Brewster angle direction.

6. Distribution of Disorder Parameters

Fig. S4. Structural distribution profiles under different disorder parameters.

7. Statistical validation of the Brewster-angle transmission channel under layer-thickness disorder

Following statistical approaches previously employed to evaluate disorder robustness in photonic systems through ensembles of independently generated disorder realizations [3, 4], we performed a realization-resolved statistical analysis of the Brewster-angle transmission channel under layer-thickness disorder。

The multilayer contains 400 nominal unit cells, and the thickness of each nominal layer segment in the $s$-th realization was independently perturbed according to

$$d_{j}^{(s)}=d_{j}^{(0)}[1+D\xi_{j}^{(s)}]$$

where $d_{j}^{(0)}$is the nominal thickness, $D$denotes the disorder strength, and$\xi_{j}^{(s)}$is independently sampled from a uniform distribution over$[-1,1]$. The refractive indices were kept unchanged throughout the disorder process. Three disorder strengths,$D=20\%,50\%$and $100\%$ were considered.

For the realization-resolved angular analysis in Fig. S5(a), 100 independent disorder realizations were generated for each disorder strength. For each realization, the reflectance was calculated over the normalized-frequency range

$$0.10\leq\frac{\omega a}{2\pi c}\leq0.55$$

and averaged over frequency at each angular offset$\Delta\theta\text{=}\theta\text{-}\theta_{B}$

$$\overline{R_{s}}(\Delta\theta)=\frac{1}{N_{f}}\sum_{i=1}^{N_{f}} R_{s}(\omega_{i},\theta_{B}+\Delta\theta).$$

The solid curves in Fig. S5(a) represent the ensemble averages of the 100 independent realizations, whereas the shaded regions indicate the central 95% realization ranges, defined by the 2.5th and 97.5th percentiles of the realization-resolved responses. Thus, the shaded regions characterize the variation among different physical disorder configurations rather than the confidence interval of the ensemble mean. For all three disorder strengths, the reflectance minimum remains pinned to$\Delta\theta=0$, while substantially larger realization-dependent variations appear away from the Brewster direction. This behavior is consistent with the fact that layer-thickness disorder modifies the propagation phases accumulated inside the multilayer but does not alter the Brewster condition itself, which is determined by the refractive-index contrast.

To further examine the statistical convergence with ensemble size, we additionally generated$N_{phys}=2000$independent disorder realizations for each disorder strength. Because a full angular scan is not required for this analysis, only the Brewster direction and the two symmetric off-Brewster control directions,$\theta_{B}-5^{\circ}$ and$\theta_{B}+5^{\circ}$, were calculated. For each realization, we define the frequency-averaged reflectance contrast as

$$\Delta R_{s}=\overline{R}_{control,s}-\overline{R}_{B,s}$$

Where

$$\overline{R}_{control,s}=\frac{\overline{R}_{s}(\theta_{B}-5^{\circ})+\overline{R}_{s}(\theta_{B}+5^{\circ})}{2}$$

A positive$\Delta R_{s}$therefore indicates a lower reflectance at the Brewster direction than at the neighboring off-Brewster directions. From the pool of 2000 independent realizations, subsets of size

$$N=5,10,20,30,40,50,60,75,100$$

were randomly sampled without replacement. For each$N$, this procedure was repeated $N_{resamp}=2000$times, and the mean reflectance contrast was calculated for every sampled subset. The solid curves in Fig. S5(b) show the mean values of these resampled ensemble estimates, while the shaded regions represent their central 95% resampling intervals. The horizontal dashed lines indicate the reference mean values obtained from the complete ensembles of 2000 independent realizations.

As the ensemble size increases, the resampling intervals progressively narrow and the estimated mean reflectance contrast becomes increasingly concentrated around the corresponding full-ensemble reference value. Together with the realization-resolved angular profiles in Fig. S5(a), these results demonstrate both the reproducibility of the Brewster-direction reflectance minimum across independent random structures and the statistical convergence of the disorder analysis. The robustness discussed here specifically refers to layer-thickness disorder with fixed refractive indices.

Fig. S5. Statistical validation of the Brewster-angle transmission channel under layer-thickness disorder.
(a) Frequency-averaged reflectance versus angular offset from the Brewster angle for 20%, 50%, and 100% disorder. Solid curves show ensemble averages over 100 independent disorder realizations, and shaded regions indicate the central 95% realization ranges.
(b) Convergence of the mean reflectance contrast$\Delta R=\overline{R}_{control}-\overline{R}_{B}$with ensemble size. For each disorder strength, 2000 independent realizations were generated. At each ensemble size, 2000 random subsets were sampled without replacement; solid curves show the mean resampled estimates, shaded regions indicate the central 95% resampling intervals, and dashed lines denote the full-ensemble means.

References

1.	P. Yeh, *Optical Waves in Layered Media* (Wiley, 2005).

2.	M. Xiao, et al., "Surface Impedance and Bulk Band Geometric Phases in One-Dimensional Systems," Physical Review X **4**, 021017 (2014).

3.	T. Dai, et al., "A programmable topological photonic chip," Nature Materials **23**, 928-936 (2024).

4.	R. Keil, et al., "The random mass Dirac model and long-range correlations on an integrated optical platform," Nature Communications **4**, 1368 (2013).
